# Depressive and Insomnia Symptoms Among Older Adults With Different Chronic Pain Trajectories: A Network Analysis Based on Observation Over an Eight-Year Period

**DOI:** 10.1155/da/8065167

**Published:** 2025-06-18

**Authors:** He-Li Sun, Pan Chen, Wei Bai, Yuan Feng, Sha Sha, Zhaohui Su, Teris Cheung, Chee H. Ng, Qinge Zhang, Yu-Tao Xiang

**Affiliations:** ^1^Unit of Psychiatry, Department of Public Health and Medicinal Administration, and Institute of Translational Medicine, Faculty of Health Sciences, University of Macau, Macao SAR, China; ^2^Centre for Cognitive and Brain Sciences, University of Macau, Macao SAR, China; ^3^Department of Epidemiology and Biostatistics, School of Public Health, Jilin University, Changchun, China; ^4^Beijing Key Laboratory of Mental Disorders, National Clinical Research Center for Mental Disorders and National Center for Mental Disorders, Beijing Anding Hospital, Capital Medical University, Beijing, China; ^5^School of Public Health, Southeast University, Nanjing, China; ^6^School of Nursing, Hong Kong Polytechnic University, Hong Kong SAR, China; ^7^Department of Psychiatry, The Melbourne Clinic and St. Vincent's Hospital, University of Melbourne, Richmond, Victoria, Australia

**Keywords:** chronic pain, depression, insomnia, longitudinal study design, older adults, pain trajectory

## Abstract

**Background:** Depressive and insomnia symptoms are common among older adults with chronic pain. We aimed to examine different chronic pain trajectories of older adults over an 8-year observation period and explore the network structures of depression and insomnia in each chronic pain group.

**Methods:** The trajectories of pain in the USA-based Health and Retirement Study (HRS) data from 2010 to 2018 were examined using latent class growth analyses (LCGA) method. Depressive and insomnia symptoms were measured with the eight-item version of the Center for Epidemiological Studies Depression (CESD-8) Scale and the four-item Jenkins Sleep Scale (JSS-4), respectively. Network models were constructed using the Ising model. Central symptoms and bridge symptoms were identified via expectedInfluence (EI) and bridge EI, respectively.

**Results:** A total of 11,132 older adults were included in the trajectory analysis, with three chronic pain trajectories identified, including “severe pain trajectory,” “moderate pain trajectory,” and “non or mild pain trajectory”. From these trajectories, “Lack of happiness” (CESD4), “Feeling depressed” (CESD1), and “Feeling sad” (CESD7) emerged as the most central symptoms, while “Feeling tired in the morning” (JSS4) was identified as the key bridge symptom. However, the findings may not be generalizable to other parts of the world outside the USA.

**Conclusion:** Older adults with different chronic pain trajectories exhibited similar depression and insomnia network structure. Implementing timely interventions that target central and bridge symptoms might mitigate the co-occurrence of depression and insomnia in this population.

## 1. Introduction

Pain, which is defined as an unpleasant sensory and emotional experience arising from actual or potential tissue damage, can be caused by numerous factors such as diseases, surgeries, and pressure injuries [1]. Based on the duration of pain, acute pain refers to pain lasting from minutes up to 3 months, while chronic pain is defined as pain persisting or recurring for 3 months or longer [2]. Globally, chronic pain is one of the most common and impactful conditions associated with considerable distress and disability, resulting in a heavy burden on the individual and health care systems [3, 4]. Chronic pain is common among older adults [5], for instance, a USA population study found a higher prevalence of chronic pain in older adults compared to younger groups [6]. Additionally, older adults are more likely to report higher pain intensity [7], with the most frequent chronic pain conditions being unspecified chronic joint pain, followed by back pain and neck pain [4]. Furthermore, older adults may perceive pain as an inevitable process of aging, which makes them less likely to report pain experiences [8]. Moreover, older adults may have apprehension about developing a potential serious illness, and thus they may be more inclined to tolerate the pain to avoid additional medical assessments, medications, or surgical interventions [9]. However, poorly controlled chronic pain among older adults can lead to a host of negative health consequences, including limited daily activity, social isolation, and loss of independence in their daily life, all of which may increase the risk of mental health problems [10].

Recent studies found that depression and insomnia often co-occur with chronic pain [10, 11]. Approximately 35% of individuals suffering from chronic pain report having depression [12], while 6%–10% of them also experience symptoms of insomnia [13]. Both depression and insomnia have a bidirectional relationship with chronic pain, i.e., severe depression/insomnia are associated with increased risk of severe pain and vice versa [13, 14]. While the close relationship between depression and insomnia with chronic pain has been amply documented in previous studies, the assessments of psychiatric problems have been largely based on total scores of assessment measures or diagnostic tools. However, it is well recognized that both depression and insomnia consist of groups of individual symptoms with different neuropsychological mechanisms [15, 16]. Therefore, it is important to examine the associations between depression and insomnia with chronic pain at a symptom level.

In recent years, network analysis has emerged as a novel approach in psychiatry and psychology to characterize psychiatric and related problems [17]. Network analysis is based on psychological network theory, in which psychiatric disorders/syndromes are viewed as dynamic systems of interacting symptoms rather than static latent constructs [18]. One of the primary advantages of network analysis is the ability to reveal relationships between individual symptoms, which overcomes the weaknesses of traditional statistical methods based on total scale scores [17]. Consequently, network analysis can identify the most central (influential) symptoms within a complex pattern of interactions and determine the relative importance of specific symptoms within a network model. Moreover, network analysis can examine important bridge symptoms, which serve as key connectors between distinct psychological clusters [19]. Identifying and addressing the bridge symptoms may potentially disrupt the co-occurrence of comorbidities [19]. Clinically, targeting the relevant central and bridge symptoms may reduce the spread of psychiatric symptoms, as their activation is strongly associated with other symptoms across the network models [18]. Network analysis has been increasingly used in psychiatry research. For example, Pan et al. [20] compared the network models of depression and insomnia symptoms between older adults with stroke and those without stroke. However, to date, no studies have explored the depression-insomnia network models among older adults with different pain trajectories. In addition, most studies that previously assessed chronic pain have used a singular question at a single time point [21, 22], rather than employing long-term studies that capture both pain trajectories as well as the complexity and variability of chronic pain experiences.

To fill these gaps, this study aimed to examine the pain trajectories of older adults based on observations over an 8-year period to explore the trends of chronic pain, evaluate the prevalence of depression and sleep problems among those with different chronic pain trajectories, and construct the network structures of depressive and insomnia network models in each pain trajectory groups to identify the most influential symptoms. Based on previous research [23, 24], we hypothesized that pain patterns and centrality features of depressive-insomnia network models would differ substantially across pain trajectory groups among older adults.

## 2. Methods

### 2.1. Study Design and Participants

This study was a secondary analysis of the Health and Retirement Study (HRS), a nationally cohort survey of adults aged 50 years and older living in the USA. The HRS is sponsored by the National Institute on Aging (grant number NIA U01AG009740) and conducted by the University of Michigan. The details of HRS are available and published elsewhere [25]. The HRS contains data on various aspects of lives through biennial assessments. In this study, we used the HRS data collected from 2010 (wave 10) to 2018 (wave 14), which were not affected by the COVID-19 pandemic and contained relatively complete data on pain, depression, and insomnia. The study timeline is shown in Figure S1. Participants were included in the study if they were 1) aged 50 years or above at wave 10 (baseline 2010); 2) completed the assessment on the pain-related questions from wave 10 to wave 14; and 3) completed the assessment on depressive and insomnia symptoms at wave 14. Those who did not complete the assessments on pain, depression, and insomnia were excluded from the study sample. The sample selection procedure is shown in Figure S2. The research protocol of the HRS has been approved by the Institutional Review Board of the University of Michigan, and all participants had provided written informed consent.

### 2.2. Measurements

Self-reported pain status was assessed using two standard questions in HRS. The first question was: “Are you often troubled with pain?” A response of “no” was coded as 0, indicating the absence of pain. The second was: “How bad is the pain most of the time: mild, moderate or severe?” This question assessed the degree of pain severity, with a score of 1 representing mild pain, 2 representing moderate pain, and 3 representing severe pain. Based on the two questions, the total score of self-reported pain ranged from 0 to 3, with a higher score indicating a more severe pain.

Depressive symptoms were evaluated using the self-reported eight-item version of the Center for Epidemiological Studies Depression (CESD-8) Scale [26], which has been widely used in assessing depressive symptoms in older adults [27]. The CESD-8 items include: “Feeling depressed”, “Everything was an effort,” “Sleep restless,” “Lack of happiness,” “Loneliness,” “Not enjoying life,” “Feeling sad,” and “Inability to get going” [27]. Two items, “Lack of happiness” and “Not enjoying life” are reverse scored. Each CESD-8 item was coded as “1” or “0”, indicating the presence or absence of specific depressive symptoms, respectively. The sum score of the CESD-8 ranged from 0 to 8, with a higher score indicating greater severity of depressive symptoms.

Insomnia symptoms were assessed using the validated self-reported four-item Jenkins Sleep Scale (JSS-4), including “Trouble falling asleep,” “Trouble maintaining sleep,” “Early morning awakenings,” and “Feeling tired in morning” [28]. Following a previous HRS study [29], participants who reported having frequency of “most of the time” and “sometimes” were assigned “1,” while “rarely or never” were assigned 0 points. The sum score of JSS-4 ranged from 0 to 4, with a greater value indicating more severe insomnia problems.

Demographic characteristics collected included age, gender, marital status, education years, drinking and smoking history, pain medicine, cognitive score, and disease status. Corresponding questions are listed in supplemental method.

### 2.3. Statistical Analysis

#### 2.3.1. Trajectory Analysis

Chronic pain trajectories in older adults were identified with latent class growth analysis (LCGA), using *“lcmm”* package [30]. This approach assumes that there is heterogeneity in the development of pain status within the target population, which can be categorized into distinct trajectory groups based on their pain status over time [31]. Through LCGA, each older adult was classified into a trajectory based on maximum likelihood estimation. In this study, we set up four potential trajectory models, and the optimal model was chosen based on following criteria: 1) lower values of BIC or AIC; 2) closer-to-1 entropy values; and 3) at least 5% of participants in each class [31]. The demographic characteristics of each pain trajectory were compared using *ANOVA* test and *chi*-square test for continuous and categorical variables, respectively. All analysis were conducted using a two-tailed test with a significance level of 0.05.

#### 2.3.2. Network Estimation

Before conducting the network model, we identified node redundancy using the goldbricker function [32]. Node redundancy occurs when a pair of nodes share more than 75% similar edges. Redundancy can compromise the relationships between nodes in the network; thus, one of the redundant nodes was excluded prior to estimating the network model [32]. As all nodes were binary variables, the depressive and insomnia networks were estimated using the Ising model [33]. To further optimize the network model, we employed the enhanced least absolute shrinkage and selection operator (eLasso) procedure to shrink weaker potential edges within the network. Finally, only the stronger edges were left in the final network model. Depressive and insomnia network model was conducted using network method with “NetworkTools” package [34].

Node centrality was evaluated using expected influence (EI), which was calculated based on the weighted value of the edges surrounding the node. EI reflects the overall connectedness of the node; specifically, nodes with a high EI value are regarded as having strong effects on the entire network model [35]. From a clinical perspective, nodes with higher EI values may have the potential to activate other nodes within the network model and thus can be targeted for therapeutic deactivation [35]. Bridge expected influence (BEI) indicates the sum connectivity of a node with nodes from other disorders [19]. The high BEI may play a key role as a connection point between two clusters; intervention on those nodes may be beneficial for breaking the co-occurrence of the two syndromes. Both EI and BEI were calculated with *“qgraph”* package [36].

Different pain trajectory network models were compared using network comparison test (NCT) [37]. We compared networks from three aspects: 1) global strength invariance test that reflected the overall difference of centrality; 2) network invariance test that reflected the overall difference among edges; and 3) centrality/edge invariance test that reflected the difference of centrality/edge of each symptom when any difference was detected in the above overall difference tests. The R package *“Network Comparison test”* was utilized to assess the network difference [38].

The network stability encompasses both EI stability and edge-weight accuracy [39]. EI stability was calculated using case drop bootstrapping, which examined whether the order of centrality remained stable even when sampling only a proportion of original data. The stability of centrality indices was evaluated through the correlation stability coefficient (CS-coefficient), CS-coefficient larger than 0.5 was usually considered as stable [39]. Edge-weight accuracy was conducted using nonparametric bootstrapping procedure. The 95% CI of edge weight value was calculated through resampling with replacement, where narrower CIs indicating more accuracy edges (Epskamp, Borsboom, et al., [39]. The R package *“bootnet”* was utilized to estimate network stability [36].

## 3. Results

### 3.1. Basic Characteristics of Pain Trajectories

**Figure 1:**
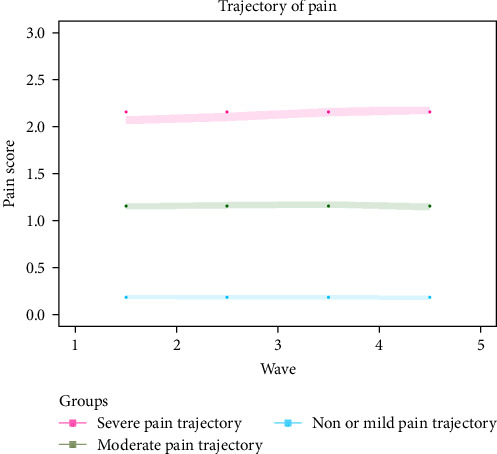
Trajectories of pain among older adults. Each color represents a chronic pain trajectory. Red color indicated severe pain trajectory (*N* = 1769); green indicated moderate pain trajectory (*N* = 2392); blue indicated non or mild pain trajectory (*N* = 6971).

A total of 11,132 participants were included for the trajectory analysis. Eventually, three chronic pain trajectories were identified (see Figure 1 and Table S1) including severe pain trajectory (*N* = 1769), moderate pain trajectory (*N* = 2392), and non or mild pain trajectory (*N* = 6971).  Table 1 illustrates the basic characteristics of participants in the 2018 wave. Compared with the other two trajectories, participants in severe pain trajectory were less likely to be male, be married, consume alcohol, and smoke, but were more likely to take opioids in the past 3 months. In terms of diseases status, participants in severe pain trajectory had a higher prevalence of hypertension, diabetes, stroke, cancer, heart condition, dementia, depression, or sleep disorder compared to the other pain trajectories (*p* < 0.05). The severe pain trajectory group also had higher mean scores in terms of CESD-8 Score, memory, executive function, and temporal orientation compared to the other trajectories (*p* < 0.05).

### 3.2. Network Structure and Their Difference

**Figure 2:**
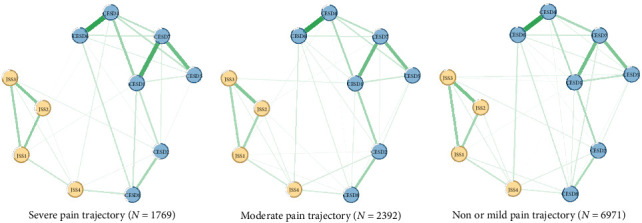
Network structure of older adults with different pain trajectories. CESD-8: Eight items of Center for Epidemiological Studies Depression; JSS-4: 4 items Jenkins Sleep Scale; CESD1: feeling depressed; CESD2: everything was an effort; CESD4: lack of happiness; CESD5: feeling lonely; CESD6: not enjoying life; CESD7: feeling sad; CESD8: inability to get going; JSS1: trouble falling asleep; JSS2: waking up during night; JSS3: waking up too early; JSS 4: feeling tired in morning.

After removing the item “Sleep” (CESD3), no redundancies were found in the network model of depressive and insomnia symptoms. The final network model comprised 11 items, including seven depressive symptoms and four insomnia symptoms.  Figure 2 depicts the network structures of depressive and insomnia symptoms between different pain trajectories. Overall, nodes from the same cluster were closely connected with each other, except for “Feeling tired in morning” (JSS4). All edges were green, indicating that the relationships between depressive and insomnia symptoms were positive. In terms of severe pain trajectory network, 31 of the 55 edges were selected into the network models, while 34 and 35 edges were left in the moderate and non-mild pain trajectories, respectively. The mean weight of edges in the severe pain trajectory was 0.388, which is lower than that in the moderate (mean weight = 0.483) and non-mild pain trajectories (mean weight = 0.499). The network invariance results are displayed in the right panel of Figure S3. No difference was found between the three pain trajectories: severe vs. non or mild (maximum difference *M*=0.708, *p*=0.31), severe vs. moderate (maximum difference *M*=0.506, *p*=0.65), moderate vs. non or mild (maximum difference *M*=0.562, *p*=0.71).

### 3.3. Network Centrality and Their Difference

**Figure 3:**
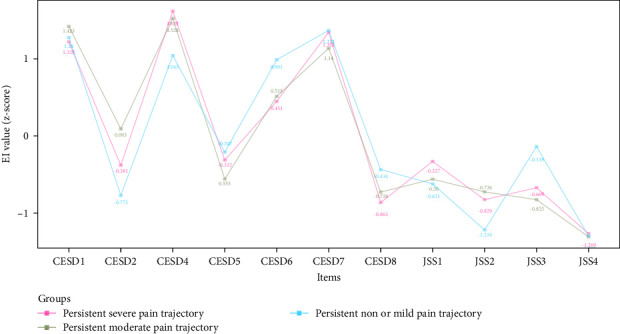
Network centrality plot of older adults with different pain trajectories. CESD-8: Eight items of Center for Epidemiological Studies Depression; JSS-4:4 items Jenkins Sleep Scale; CESD1: feeling depressed; CESD2: everything was an effort; CESD4: lack of happiness; CESD5: feeling lonely; CESD6: not enjoying life; CESD7: feeling sad; CESD8: inability to get going; JSS1: trouble falling asleep; JSS2: waking up during night; JSS3: waking up too early; JSS 4: feeling tired in morning.

**Figure 4:**
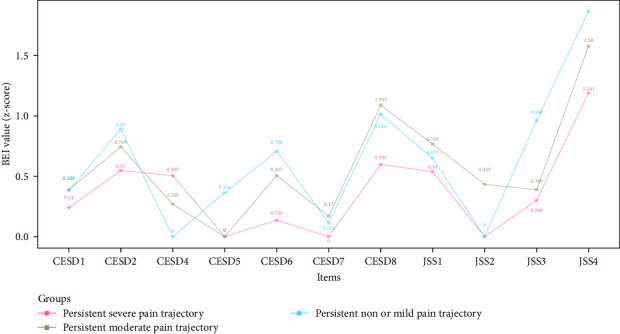
Network bridge expected influence plot of older adults with different pain trajectories. CESD-8: Eight items of Center for Epidemiological Studies Depression; JSS-4:4 items Jenkins Sleep Scale; CESD1: feeling depressed; CESD2: everything was an effort; CESD4: lack of happiness; CESD5: feeling lonely; CESD6: not enjoying life; CESD7: feeling sad; CESD8: inability to get going; JSS1: trouble falling asleep; JSS2: waking up during night; JSS3: waking up too early; JSS 4: feeling tired in morning.

Figure 3 shows the EI values of depressive and insomnia symptoms network model between different pain trajectories; each symptom shows similar EI values. Regardless of the pain trajectories, “Lack of happiness” (CESD4), “Feeling depressed” (CESD1), and “Feeling sad” (CESD7) were the most central symptoms. The global strength invariance test is shown in left panel of Figure S3. There was a significant global strength difference (*S*=6.075, *p* < 0.001) between severe and non or mild pain trajectories. The centrality invariance result is displayed in Table S3. We found the EI values of “Everything was an effort” (CESD2), “Inability get going” (CESD8), and “Waking up too early” (JSS3) were significantly different between severe vs. non or mild pain trajectories (*p* < 0.05). In addition, no global centrality difference was found in severe vs. moderate (*S*=2.709, *p*=0.08) and moderate vs. non or mild (maximum difference *S*=3.365, *p*=0.32). As for the BEI shown in Figure 4, “Feeling tired in morning” (JSS4) was the symptom with highest BEI value. The details of EI and BEI values are shown in Table S2.

### 3.4. Network Stability

Figure S4 shows that CS-coefficients of EI and BEI were 0.75 in both non or mild pain and moderate pain trajectory. In severe pain trajectory, the CS-coefficients of EI and BEI were 0.672 and 0.595, respectively. All CS-coefficients were larger than 0.5, indicating the EI and BEI of three networks were stable. Figure S5 shows a narrow range of bootstrapped 95% CIs for estimated edge weights, indicating that the edges of networks were accurate.

## 4. Discussion

To the best of our knowledge, this was the first network analysis study that examined the inter-relationships between depressive and insomnia symptoms in older adults with different chronic pain trajectories. Three chronic pain trajectories were identified, including “severe pain trajectory,” “moderate pain trajectory,” and “non or mild pain trajectory”. Based on these trajectories, “Lack of happiness” (CESD4), “Feeling depressed” (CESD1), and “Feeling sad” (CESD7) emerged as the most central symptoms, while “Feeling tired in the morning” (JSS4) was identified as the key bridge symptom.

In this study, we identified three distinct chronic pain trajectories among older adults. Specifically, 15.9% of the participants followed the severe pain trajectory, 21.5% followed the moderate pain trajectory, while 62.6% were in the non or mild pain trajectory. Chronic pain is a common condition among the older population [8, 40–42]; however, to date, there is lack of research quantifying the severity of chronic pain experienced by this population. For instance, according to a systematic review of pain [8], 60%–75% of people over the age of 65 years experienced at least “some persistent pain”. A large-scale survey of 49.7 million older people conducted across five countries (United Kingdom, France, Spain, Germany, and Italy) found that among older adults who reported pain, ~60% had “moderate” pain and 25% had “severe” pain [41]. The prevalence of pain was also influenced by the differences in study sample. For instance, the degree of pain was reported as higher among nursing home residents than in community-dwelling older adults [43]. As mentioned previously, as some older adults might be hesitant to report pain symptoms, it is crucial for healthcare professionals to pay attention to pain management, particularly among those residing in nursing homes or hospitals [44]. Furthermore, as all three chronic pain trajectories remained stable over the 8-year period, this suggest that older adults tended to experience a stable level of pain over time. This is consistent with previous research showing that the majority of people in the general population with chronic pain had stable pain trajectories over time [45].

In the depression and insomnia symptom network models of older adults, “Lack of happiness” (CESD4), “Feeling depressed” (CESD1), and “Feeling sad” (CESD7) emerged as the most influential symptoms, regardless of the type of chronic pain trajectories. All the top three most central symptoms came from the depression community, suggesting that depressive symptoms play a key role in maintaining and activating the co-occurrence of depression and insomnia. Similar to our findings, a previous network study on depressive and insomnia symptoms in older adults with and without cancer also identified “Feeling sad,” “Feeling depressed,” and “Lack of happiness” as the most prominent symptoms in both groups [46]. Additionally, another network analysis study on depressive and insomnia symptoms among older adults with stroke yielded comparable results, revealing that “Feeling depressed” and “Feeling sad” were the two most central symptoms in older adults with stroke [46]. A possible explanation might be that “Lack of happiness” (CESD4) could be closely related to the inability to experience enjoyment or pleasure [47], while “Feeling depressed” (CESD1) and “Feeling sad” (CESD7) might be aligned with “depressed mood”. In the diagnostic criteria for major depressive disorder (MDD), the symptoms of “depressed mood” and “loss of interest or pleasure” are among the fundamental symptoms of this disorder [48]. As such, targeting the fundamental depressive symptoms might be beneficial for controlling not only depression but also comorbid insomnia among older adults. The centrality of certain items (e.g., “Not enjoy life” (CESD6), “Inability get going” (CESD8), “Waking up too early” (JSS3)) showed small differences between severe and non-mild pain trajectories. However, these items did not rank high in the centrality of depression and insomnia network, suggesting that they had limited influence on the activation of the network model.

In the network models investigating three pain trajectories, “Feeling tired in the morning” (JSS4) was the most important bridge symptom, suggesting its potential mediating role between depression and insomnia in older adults. “Feeling tired in the morning” (JSS4) refers to waking up with fatigue and exhaustion, which relates to daytime symptoms compared with other three JSS nighttime symptoms [49]. Older adults who have shorter sleep duration often report feeling tired in the morning [49]. Therefore, prolonging the sleep duration of older adults might effectively improve their morning energy levels and potentially mitigate the development of depressive symptoms.

From a clinical perspective, our findings could provide effective strategies for managing depression-insomnia in older adults with chronic pain. Timely interventions targeting core depressive symptoms, such as “Lack of happiness,” “Feeling depressed,” and “Feeling sad,” could help reduce the progression of developing comorbidities of depression and insomnia. For instance, promoting lifestyle modifications such as physical activity, social engagement, and mindfulness exercises might enhance emotional resilience and reduce depressive symptoms in those with moderate-to-severe pain trajectories [50, 51]. Additionally, recognizing the onset of bridge symptoms like “Feeling tired in the morning” (JSS4) could serve as early indicators of depressive symptoms. Activity routines after awakening could disrupt the pathway linking insomnia to depression by addressing this bridging symptom [52, 53].

This study had several strengths. First, we utilized a large sample size, which enhanced the statistical power of the findings. Additionally, we used a long observational period spanning over 8 years, which enabled the characterization of chronic pain, providing valuable insights into its dynamic changes. Finally, the utilization of network analysis enabled the exploration of the most influential central symptoms and unique associations among symptoms. However, several limitations should be noted. First, the assessment of depression and insomnia relied on self-reported rather than objective measurements, which could result in recall bias. Second, the study lacked data on the cause, site, and type of pain, limiting the ability to evaluate the differences in specific pain subgroups such as those with cancer pain or neuropathic pain, or others. Third, the findings of this study could not be generalized to other countries/regions due to the exclusive focus on older adults in the USA. Fourth, given the observational design of the study, causal relationship between depression and insomnia could not be assessed. Finally, despite efforts to control confounding factors, other unaccounted factors might still influence the relationships between pain trajectory, depression, and insomnia.

In conclusion, despite differences in the prevalence of depression and insomnia among older adults with different chronic pain trajectories, the core symptoms of depression and insomnia (“Lack of happiness," “Feeling depressed," and “Feeling sad") remained consistent over time. In addition, “Feeling tired in the morning” emerged as the most influential bridging symptom. Evidence-based psychosocial interventions that target these key symptoms might be beneficial for addressing comorbid depression and insomnia among older adults with different pain trajectories.

## Figures and Tables

**Table 1:** Characteristics of participants (*N* = 11,132).

Characteristics	Severe pain trajectory (*N* = 1769)	Moderate pain trajectory (*N* = 2392)	Non or mild pain trajectory (*N* = 6971)	*p* Value
	*N* (%)			
Male	535 (30.2)	882 (36.9)	3013 (43.2)	<0.001
Married	808 (45.7)	1278 (53.4)	4159 (59.7)	<0.001
Alcohol consumption	770 (43.5)	1264 (52.8)	4126 (59.2)	<0.001
Smoke	1112 (62.9)	1374 (57.5)	3559 (51.1)	<0.001
Took opioids in the past 3 months	636 (36.0)	403 (16.8)	304 (4.4)	<0.001
Hypertension	1376 (77.8)	1668 (69.7)	4141 (59.4)	<0.001
Diabetes	674 (38.1)	765 (32.0)	1745 (25.0)	<0.001
Stroke	239 (13.5)	197 (8.2)	448 (6.4)	<0.001
Cancer	343 (19.4)	460 (19.2)	1158 (16.6)	0.023
Heart condition	698 (39.5)	743 (31.1)	1533 (22.0)	<0.001
Alzheimer's disease	13 (0.7)	24 (1.0)	40 (0.6)	0.278
Dementia	77 (4.4)	45 (1.9)	98 (1.4)	<0.001
History of depression	935 (52.9)	752 (31.4)	1095 (15.7)	<0.001
History of sleep disorder	503 (28.4)	459 (19.2)	774 (11.1)	<0.001
	Mean (SD)			
Age, mean	75.02 (9.49)	76.05 (9.86)	75.95 (9.91)	<0.001
Education years	12.15 (3.09)	12.73 (3.13)	13.21 (3.12)	0.823
CESD-8 score	2.81 (2.43)	1.82 (2.12)	0.88 (1.47)	<0.001
Memory score	9.35 (3.35)	9.89 (3.50)	10.19 (3.59)	<0.001
Executive function score	4.77 (1.97)	5.04 (1.98)	5.20 (1.97)	<0.001
Temporal orientation score	3.68 (0.60)	3.71 (0.56)	3.72 (0.57)	0.077

*Note:* CESD-8: Eight items of Center for Epidemiological Studies Depression; alcohol: self-reported history of having any alcoholic beverages such as beer, wine, or liquor. Smoke: self-reported history of having ever smoked; History of depression and insomnia were based on diagnosis of doctor or nurse.

## Data Availability

The data could be access through https://hrs.isr.umich.edu/.

## Supporting Information

Supporting Information Figure S1. Timeline and study design. Figure S2. Flow chart of inclusion of the study population. Figure S3. Network comparison. Figure S4. Bootstrapped stability. Figure S5. Edge-weight confidence interval. Table S1. Estimated coefficients for the models estimated by latent class growth analyses (LCGA). Table S2. Node labels and item descriptions. Table S3. Network centrality invariance test between severe pain and non or mild pain trajectory.
