# Supplementary material for: Depressive and Insomnia Symptoms Among Older Adults With Different Chronic Pain Trajectories: A Network Analysis Based on Observation Over an Eight-Year Period
Source: Depress Anxiety. 2025 Jun 18;2025:8065167. doi: 10.1155/da/8065167 (PMC12197484; doi:10.1155/da/8065167)

**Supplement materials**

**Figure S1. Timeline and study design**

**Figure S2. Flow chart of inclusion of the study population**

**Figure S3. Network comparison**

**Figure S4. Bootstrapped stability**

**Figure S5. Edge-weight confidence interval**

**Table S1. Estimated coefficients for the models estimated by Latent Class Growth Analyses (LCGA)**

**Table S2. Node labels and item descriptions**

**Table S3. Network centrality invariance test between severe pain and non or mild pain trajectory**

**Supplement method**

Memory was measured using immediate and delayed word recall tests, in which participants needed to recall 10 unrelated words immediately or after a certain period, and each correct answer was assigned with 1-point, with the total memory score ranging from 0 to 20.

Executive function was measured using counting backwards test and serial sevens test. In the counting backwards test, participants were asked to count backwards for continuous numbers from 20. If they answered correctly on their first try, they were assigned 2 points; for the second try, 1 point was assigned if answered correctly, and 0 points were assigned if they were unable to answer correctly on either attempt. In serial sevens test, participants were asked to subtract 7 successively from 100 five times, with the total executive function score ranging from 0-7.

Orientation was assessed using four-time related questions, regarding the day of month, month, year, and day of week. 1 point was given to each correct answer, giving the total score of 0-4.

**Figure S1. Timeline and study design**

**
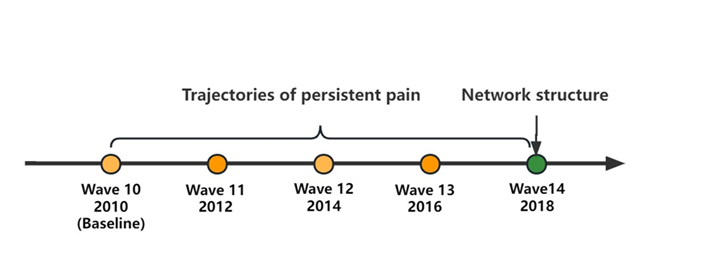
**

**Figure S2. Flow chart of inclusion of the study population**

non or mild pain trajectory

(N=6,971)

moderate pain trajectory

(N=2,392)

Excluded:

1. Missing depression or insomnia data

11,864 participants aged ≥50 who responded regarding pain level from wave 10 to wave14

11,132 participants

severe pain trajectory

（N=1,769)

**Table S1. Estimated coefficients for the models estimated by Latent Class Growth Analyses (LCGA)**

| **Groups** | **AIC** | **BIC** | **Entropy** | **Class1 (%)** | **Class2 (%)** | **Class3 (%)** | **Class4 (%)** |
| --- | --- | --- | --- | --- | --- | --- | --- |
| 1 | 167883.2 | 167905.4 | 1.00 | 100 |  |  |  |
| 2 | 141481.9 | 141526.1 | 0.9219575 | 28.02 | 71.98 |  |  |
| 3 | 137508.0 | 137574.4 | 0.8681786 | 21.62846 | 62.35671 | 16.01483 | NA |
| 4 | 137514.0 | 137602.6 | 0.5933881 | 0.00 | 61.40425 | 16.01483 | 22.58092 |

**Table S2. Node labels and item descriptions**

| **Nodes** | **Label** | **Severe pain trajectory**  **(N=1,769)** | | **Moderate pain trajectory**  **(N=2,392)** | | **Non or mild pain trajectory (N=6,971)** | |
| --- | --- | --- | --- | --- | --- | --- | --- |
|  |  | **EI** | **BEI** | **EI** | **BEI** | **EI** | **BEI** |
| **Depressive symptoms** | | | | | | | |
| Feeling depressed | CESD1 | 1.225 | 0.240 | 1.423 | 0.389 | 1.281 | 0.386 |
| Everything was an effort | CESD2 | -0.381 | 0.550 | 0.093 | 0.745 | -0.772 | 0.890 |
| Lack of happiness | CESD4 | 1.622 | 0.505 | 1.528 | 0.269 | 1.045 | 0.000 |
| Loneliness | CESD5 | -0.312 | 0.000 | -0.555 | 0.000 | -0.207 | 0.366 |
| Not enjoying life | CESD6 | 0.451 | 0.136 | 0.515 | 0.507 | 0.991 | 0.708 |
| Feeling sad | CESD7 | 1.352 | 0.000 | 1.140 | 0.170 | 1.371 | 0.115 |
| Inability get going | CESD8 | -0.863 | 0.599 | -0.728 | 1.093 | -0.434 | 1.016 |
| **Insomnia symptoms** | | | | | | | |
| Trouble falling asleep | JSS1 | -0.327 | 0.540 | -0.560 | 0.768 | -0.621 | 0.651 |
| Waking up during night | JSS2 | -0.829 | 0.000 | -0.726 | 0.435 | -1.219 | 0.000 |
| Waking up too early | JSS3 | -0.669 | 0.299 | -0.825 | 0.389 | -0.138 | 0.961 |
| Feeling tired in morning | JSS4 | -1.269 | 1.191 | -1.306 | 1.580 | -1.299 | 1.870 |
| Notes: CESD-8: Eight items of Center for Epidemiological Studies Depression; JSS: Jenkins Sleep Scale; EI: Expected Influence (z-score); BEI: Bridge Expected Influence (1-step) | | | | | | | |

**Figure S3. Network comparison**

**
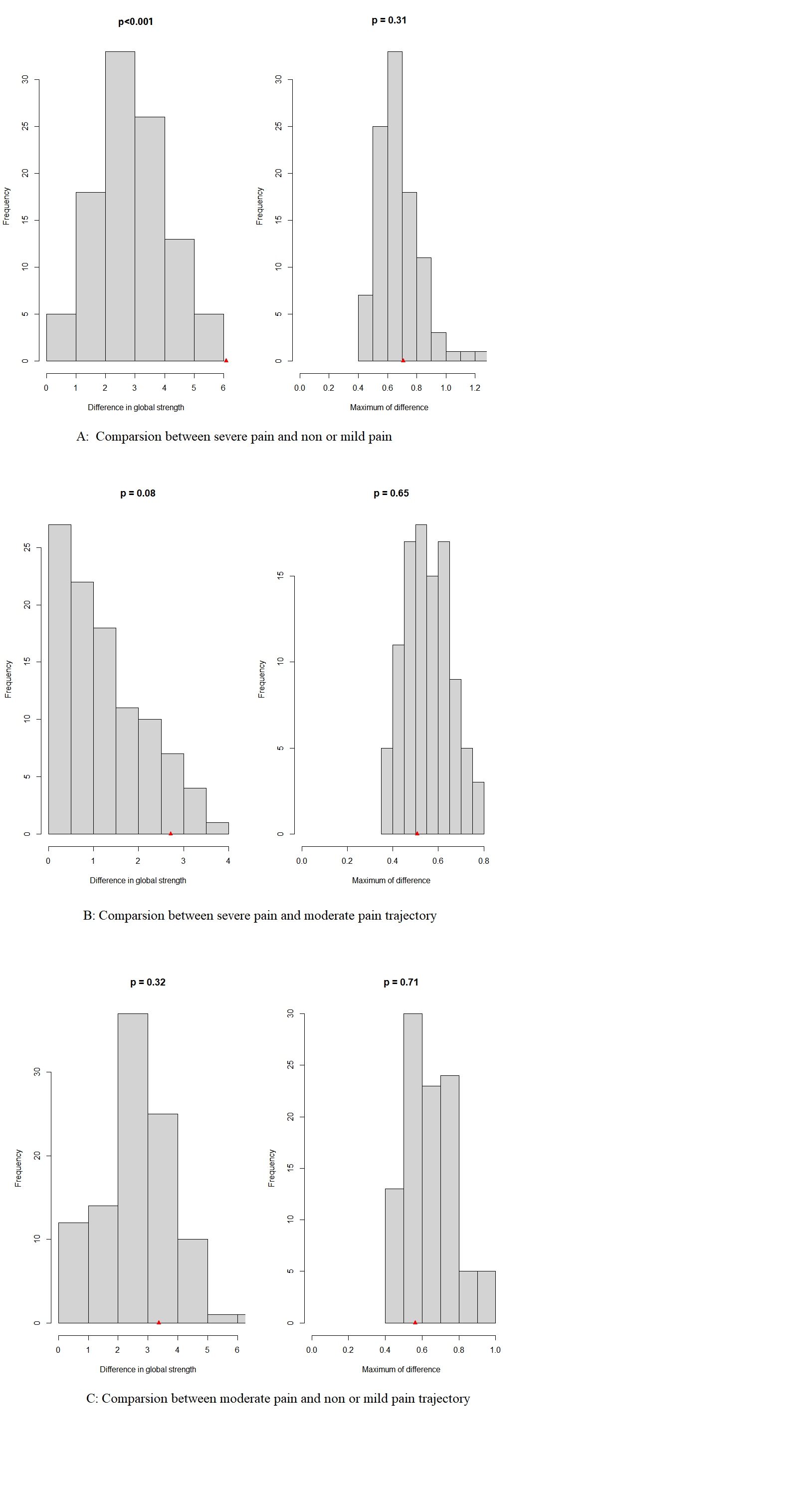
**

**Table S3. Network centrality invariance test between severe pain and non or mild pain trajectory**

| **Nodes** | **Label** | **p** |
| --- | --- | --- |
| **Depressive symptoms** | | |
| Feeling depressed | CESD1 | 0.14 |
| Everything was an effort | CESD2 | 0.34 |
| Lack of happiness | CESD4 | 0.84 |
| Loneliness | CESD5 | 0.26 |
| Not enjoying life | CESD6 | **0.03** |
| Feeling sad | CESD7 | 0.32 |
| Inability get going | CESD8 | **<0.001** |
| **Insomnia symptoms** | | |
| Trouble falling asleep | JSS1 | 0.38 |
| Waking up during night | JSS2 | 0.21 |
| Waking up too early | JSS3 | **0.01** |
| Feeling tired in morning | JSS4 | 0.10 |

**Figure S4. Bootstrapped stability**


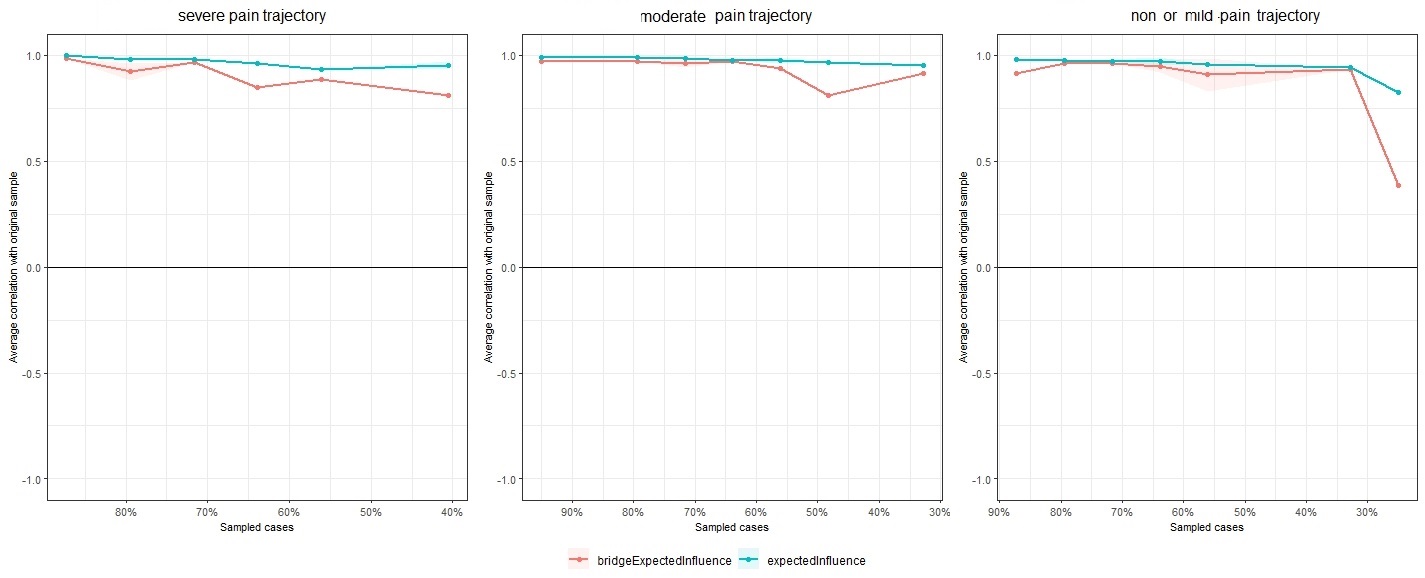


**Figure S5. Edge-weight confidence interval**


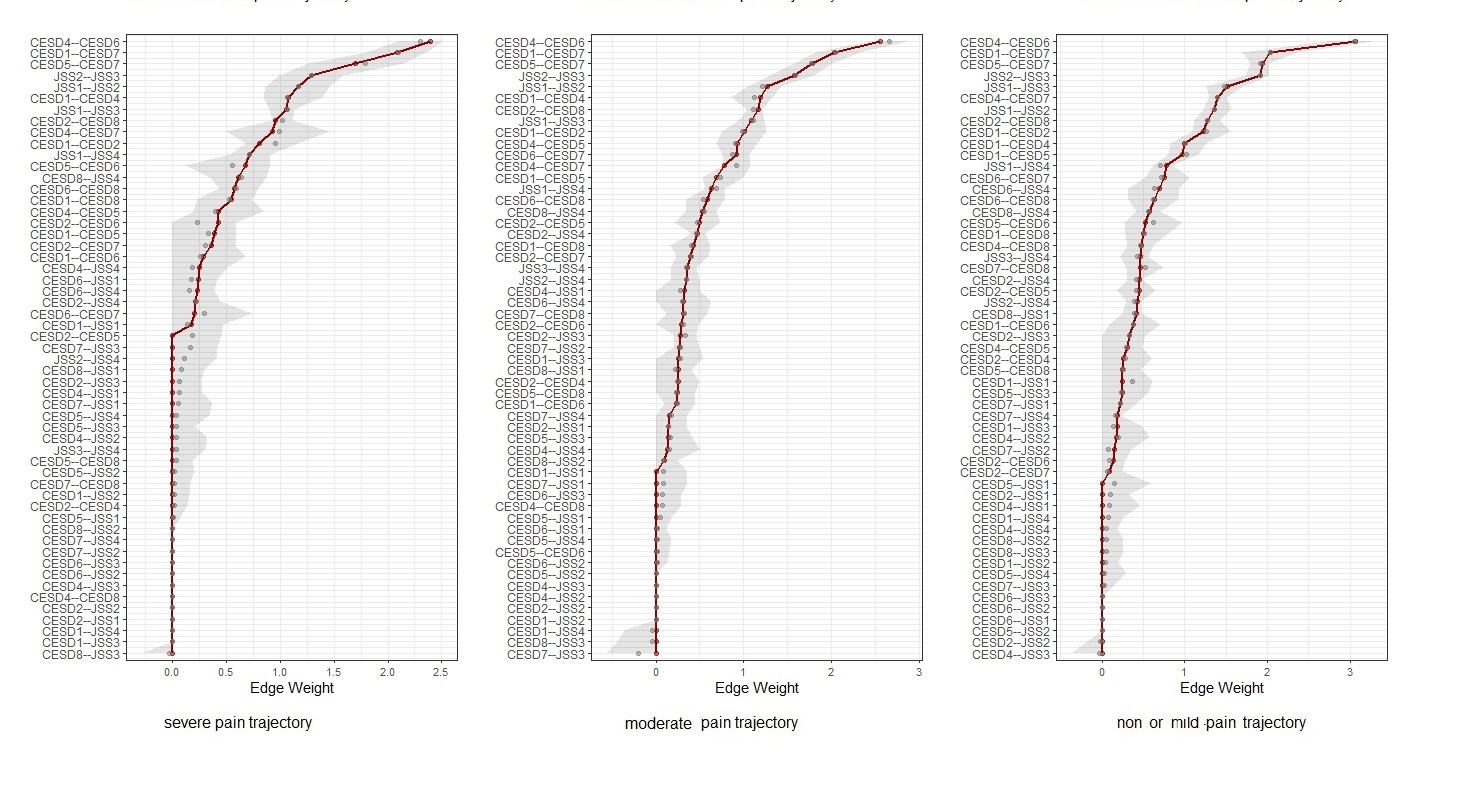

Supplement: Supporting Information — Figure S1. Timeline and study design. Figure S2. Flow chart of inclusion of the study population. Figure S3. Network comparison. Figure S4. Bootstrapped stability. Figure S5. Edge-weight confidence interval. Table S1. Estimated coefficients for the models estimated by latent class growth analyses (LCGA). Table S2. Node labels and item descriptions. Table S3. Network centrality invariance test between severe pain and non or mild pain trajectory. [file 8065167.f1.docx]
